# Shifting boundaries, extended minds: ambient technology and extended allostatic control

**DOI:** 10.1007/s11229-025-04924-9

**Published:** 2025-02-06

**Authors:** Ben White, Andy Clark, Avel Guènin-Carlut, Axel Constant, Laura Desirée Di Paolo

**Affiliations:** 1 School of Media, Arts and Humanities, University of Sussex, Brighton, UK; 2 School of Engineering and Informatics, University of Sussex, Brighton, UK; 3 School of Psychology, Children and Technology Lab, University of Sussex, Brighton, UK

**Keywords:** Extended mind, Active inference, Ambient technology, Allostasis, Cognitive niche construction

## Abstract

This article applies the thesis of the extended mind to ambient smart environments. These systems are characterised by an environment, such as a home or classroom, infused with multiple, highly networked streams of smart technology working in the background, learning about the user and operating without an explicit interface or any intentional sensorimotor engagement from the user. We analyse these systems in the context of work on the “classical” extended mind, characterised by conditions such as “trust and glue” and phenomenal transparency, and find that these conditions are ill-suited to describing our engagement with ambient smart environments. We then draw from the active inference framework, a theory of brain function which casts cognition as a process of embodied uncertainty minimisation, to develop a version of the extended mind grounded in a process ontology, where the boundaries of mind are understood to be multiple and always shifting. Given this more fluid account of the extended mind, we argue that ambient smart environments should be thought of as extended allostatic control systems, operating more or less invisibly to support an agent’s biological capacity for minimising uncertainty over multiple, interlocking timescales. Thus, we account for the functionality of ambient smart environments as extended systems, and in so doing, utilise a markedly different version of the classical thesis of extended mind.

## Introduction

According to the Thesis of the Extended Mind (TXM), some genuinely cognitive operations can be partly constituted by activities involving material tools, artefacts, and devices (Clark & Chalmers, 1998). When the fit is right, and certain conditions met, such external devices and human biological cognition can form an integrated system—an ‘extended mind’ (Clark, 2008, 2010; Clark & Chalmers, 1998). This is a strong thesis stating that the substrate of mind can, literally, lie in external materiality. In the years since the original (1998) paper was published, spirited debate has surrounded what that right fit, and what those conditions are. Thus far, these debates have focused almost entirely on devices, tools, and artefacts, like notebooks (Clark & Chalmers, 1998), smartphones (Bruineberg, 2023; Bruineberg & Fabry, 2022; Chalmers, 2008), laptop computers (Carter & Palermos, 2016), video game interfaces (Kirsh, 2019; Kirsh & Maglio, 1994), and navigation systems (Clowes, 2020; Wheeler, 2019), as well as science fiction examples like the replacement of neural circuitry with silicon circuits (e.g. Clark, 2008; Clark & Chalmers, 1998). New forms of technology are emerging though, and this article focuses on the role that ambient smart technology could come to play in daily life. Recent work has brought into focus ambient smart environments, highlighting the conceptual differences between such systems and the technology that has featured in most of the TXM literature. It’s argued that ambient smart environments exhibit a radical functionality that sees them playing an “upstream” role in attention and action selection (Verbeek, 2009; Aydin et al., 2019; White & Miller, 2024; White & Hipólito, 2024; Hipólito & White, 2023). In this article, we assess the applicability of TXM to ambient smart environments, arguing that they present a challenging case for classical formulations of TXM. We introduce the active inference framework, with a two-fold goal: first, demonstrating that an active inference reading of TXM supports a more fluid and process-orientated form of TXM, that is more able to make sense of emerging cases like ambient technology. And second, such an account leads to the view that such ambient systems can indeed form extended cognitive systems, which we cast as a form of extended allostatic control.

In Section One we introduce TXM, highlighting some canonical examples and concerns. In Section Two we introduce ambient smart environments, providing a vivid example. In Section Three, we ask how ambient smart environments fare against the conditions of classical TXM, asking whether ambient smart environments should count as extended systems. In the final two sections (Sec. 4 & 5), we introduce active inference, seeing how it might change the way we conceptualise TXM, and think again about ambient smart environments.

In summary, our view is that ambient smart environments should be viewed as *extended allostatic control* systems, extending an agent’s capacity for optimal planning and anticipatory action. Moreover, we suggest that ambient smart environments highlight the way that it’s likely to become increasingly clumsy to continually draw a sharp line exactly where an extended cognitive system stops and the rest of the (external) machinery begins. While in many contexts it will still be useful and important to use sensible conditions to set down a line of demarcation, we show that active inference helps facilitate a different approach that conceptualises our coupling with external technological resources as rolling processes rather than fixed states, where humans and technology can form transient shifting “ecosystems of intelligence” (Friston et al., 2022). These shifting ecosystems see our biology nested within interlocking circles of bio-external machinery with shifting grades of integration, autonomous agency, and trust.

## The extended mind

### Classical TXM

The thesis of the extended mind (TXM) holds that under certain conditions, some external, material substrates supporting cognitive processes and mental states can be considered as part of the mind.^1^ At the heart of TXM sits the often misunderstood Parity Principle, which goes like this:

If, as we confront some task, a part of the world functions as a process which, were it to be done in the head, we would have no hesitation in recognising as part of the cognitive process, then that part of the world is (so we claim) part of the cognitive process (Clark & Chalmers, 1998. Pp. 8)

The point of the principle is to undermine the temptation to focus too much on the character of biological processing when making assessments about what constitutes a cognitive system. The Parity Principle says nothing at all about any fine-grained similarities between the processes themselves, though. It is not about identifying neural or bodily processing and then looking outward to see if something external can be found to be doing the same kinds of processing. In other words, the Parity Principle speaks to a more coarse-grained parity of *opportunity*—to what the functional assembly allows us to achieve—asking whether, were that to be achieved using only bio-internal resources, we would call it cognitive.

The classic example concerns Otto and Inga, who are making their way to the Museum of Modern Art in New York (Clark & Chalmers, 1998). Inga thinks and recalls (i.e., retrieves from biological memory) that the MOMA is on 53rd Street, and so she goes to see the exhibition. This is taken to be an indubitable case of a dispositional belief supported by internal bio-memory that can be called upon to navigate effectively. Otto is also on his way to see the exhibition at the MOMA, but Otto suffers from a mild case of Alzheimer’s disease. Fortunately, Otto is in the habit of writing down useful information in his notebook, which he has with him all the time, and in the notebook is the address of the MOMA. After consulting his notebook, Otto quickly forms the intention to travel to 53rd Street, and makes his way to the museum. According to TXM, Otto should be counted as holding (even prior to consulting the notebook) the correct dispositional belief too. The notebook passes the parity test because if something inside the head played the same coarse-grained functional role (that of enabling the right kind of occurrent mental state on demand) we would have no hesitation in counting the notebook as part of Otto’s cognitive machinery.

### Constraining TXM

Despite this being the canonical and very widely cited example case of extended mind, some have pushed back, arguing that while extended cognition is in principle possible, the case of Otto should not be counted (e.g., Kirsh, 2019). Indeed, Ned Block has stated that while TXM was false when the paper was written, it has since come true with the widespread use of smartphones (quoted in Chalmers, 2019: Pp.12). Questions about what kind of brain-to-technology couplings should count as cases of extension have spawned discussion about what conditions might apply. Suggested criteria for being part of an extended cognitive system include that the resource be reliably invoked, that the retrieved information is more or less automatically endorsed, that it’s easily accessible (the so called “trust and glue” criteria) (Clark, 2008, 2010; Clark & Chalmers, 1998), that the coupling be fast and continuous, phenomenologically transparent (Kirsh, 2019; Thompson & Stapleton, 2009), and reciprocally bidirectional (Kirsh, 2019), and that a subject be, in some sense, epistemically responsible for the contents of a non-neural resource (Roberts, 2012). Kim Sterelny (2010) describes how a select few resources which are “importantly, robustly, reliably or persistently supportive of decision making” could be identified as cognitive extension (Sterelny, 2010. Pp. 466).

### Expanding TXM

While some have offered constraints for TXM, others have applied its core principle to a wider array of mental phenomena. For instance, it’s been argued that if “purely cognitive” phenomena are amenable to extension, then so should our affective and emotional states (Columbetti & Roberts, 2015; Krueger & Szanto, 2016), and that our autobiographical memories can be materially extended (Heersmink, 2020). Cases have been made for extended personhood (Clowes, 2020; Heersmink, 2016), creativity (Wheeler, 2018), and for extended moral agency (Heersmink, 2017). Others have argued that consciousness itself is extended (Kirchhoff & Kiverstein, 2019; Vold, 2020). Joseph Heath and Joel Anderson (2010) have argued that “the will” extends, based on the observation that reorganising material space can introduce friction or smoothness that modulates the difficulty of doing hard things that, on some level, we might not want to do.

A detailed exploration of the TXM literature and its wide-ranging impact is beyond the scope of this article, but it’s important to see that the limits of the applicability of TXM continue to be fought over. Arguments about what should or should not count, and by which criteria we are to decide, are ongoing. This is important because, as we’ll see, emerging forms of technology might invite us to reconsider these conditions, even where they’re seemingly well established.

## Ambient smart environments

### Introducing ambient smart environments

In the near future humans will inhabit environments that are increasingly adaptive to their needs. Research in “ubiquitous” or “pervasive” computing has continued and developed a vision first introduced by Mark Weiser (1993), of environments infused with information processing technology, where the traditional user interface dissolves. Weiser’s vision was of environments infused with computing power wherein the user does not have to intentionally interact with a computing system. Computing technology will be embedded into the material of everyday things, like walls, floors, tables, and even the paint on the wall (Heylen, 2012).

These ubiquitous and invisible systems will perform their function without the need for intentional user input: “use as a conscious process will not apply to twenty-first century, ‘smart’, ambient technologies” (Brenner, 2007). In this sense, we can say that the pragmatic function of such technology, such as automatically adjusting the temperature settings on the thermostat, *precedes* the user. A low-tech example of such pragmatic precedent is the modern car key fob that opens the car when the owner walks close. Wherever we describe the functionality of ambient technology as “passive”, we are referring to this form of pragmatic precedent.

But new technological developments in machine learning mean that systems will not precede the user only pragmatically, but also *epistemically.* New smart capabilities realised in various kinds of sensors and wearables, for instance, could allow systems to gather deep reservoirs of data about the user.^2^ Ambient smart environments (ASEs) are local environments, such as classrooms or homes, permeated with this kind of pervasive and invisible technology. A smart bathroom alone could monitor gum health through a toothbrush, weight fluctuations through hidden floor scales, signs of eye problems like glaucoma using mirror-gazing, the composition of waste via a smart toilet. It could track trends in users’ physical activity, the quality of sleep, medium to long-term trends in productivity and work, changes in environmental organisation, and behavioural changes in the carrying out of everyday tasks that might warrant analysis (Cicirelli et al., 2021). Crucially, these systems will be in the position to identify important causal dynamics that the users are unlikely to be able to identify, due to their obscurity and intrinsic complexity. The aetiology of different kinds of health complaints, for instance, that are often difficult (or impossible) to track, given the kaleidoscopic array of potential causal factors in the modern world. The insight of such systems may well be far deeper than that of even the most perceptive and self-reflective individuals (or, indeed, human experts) (White & Miller, 2024; White & Hipólito, 2024; Hipólito & White, 2023). Current smart technologies, such as apps running on our smartphones, already monitor certain behaviours, learning what we like and what interests us. To make better predictions and suggestions these technologies use algorithms that interact with users in useful and creative ways, often by making suggestions or curating content (Lazer et al., 2021).

One important question to ask regarding ASEs is: what are they actually doing? One approach for thinking about human-technology interaction is thinking in terms of affordances (e.g. Norman, 2013). Affordances here are understood as *opportunities for actions* that emerge from a relation between an agent and the environment (Chemero, 2003; Gibson, 1979; Heft, 1989). But when the whole point of the functionality of the system is for the user to not have to *do* anything, it’s difficult, if not impossible, to describe that system in terms of affordances (White & Miller, 2024). Arguing that an affordance-based framework is ill-suited when applied to ASEs, recent work has claimed that the best way to understand the functionality of ASEs is as operating “upstream” of affordances, sculpting the agent’s affordances in real time (White & Miller, 2024).

According to this account, we can think of these forces as shaping an agent’s *field of affordances*, which is to say, the affordances that stand out to the agent as soliciting ​​(Bruineberg & Rietveld, 2014). For example, while my broader landscape of affordances is broad and mostly static, certain temporary constraints (of many different kinds) will mean that some of those stand out as more relevant or inviting than others in a way that’s almost constantly changing. For instance, right now my laptop and coffee cup seem inviting, but an hour from now the bathroom might exert a strong pull. The field of affordances is typically shaped by both the intrinsic dynamics of the agent, and by an array of external contextual influences (van Dijk & Rietveld, 2017). This active shaping of the field of affordances, through a hidden, adaptive, and upstream functionality is, we think, an apt description of the function of ASEs.

### Living in an ASE

Here, an example will help: consider Utto, who works from home but finds life quite stressful. The anxiety that he experiences is also impacting his sleep and diet; stress seems to make effort in other areas impossible and haunt him when he goes to bed. A combination of wearable technologies and sensors around the home begin collecting physiological, behavioural, and environmental data. Over time, the system starts to build a causal generative model reflecting deep regularities and structures that Utto himself could never track. The system then makes some more or less subtle interventions. Based on real-time data the system can adaptively manage workflow. For example, it will learn when Utto most struggles with stress, when he’s likely to procrastinate, monitoring blood glucose levels to predict dips in energy before they happen, and then shifting tasks around, modulating complexity to match Utto’s state. For example, when Utto is busy and focused, the system could slow down the rate of incoming emails, perhaps blocking non-urgent incoming contact altogether (Verbeek, 2009). In its text-based feedback and support, the system will make use of large language models to present information using figurative language to help shape thought patterns and influence mood and behaviour (e.g., Thomley & Safron, 2021; Kukkonen, 2020).

More impressively, the system may then monitor for signs of increasing variability in outward (behavioural) and inward (physiological) markers. Increases in variability are linked to prime windows for destabilising patterns of psychological ill health and are therefore opportunities for therapeutic intervention (Hanna, 1996; Hayes & Andrews, 2020; Hayes & Yasinski, 2015). The system also monitors Utto’s overall health, suggesting supplementation where it finds deficiency or necessity (Clark, 2003; Verbeek, 2009). In terms of reducing complexity, the system can ease the overall stress in Utto’s life by taking over important tasks that stressed-out Utto often neglects, and that would be likely to accumulate. Tasks like budgeting for healthy groceries, paying bills, and more of modernity’s grinding minutiae. In terms of *increasing complexity*, the system could make it more difficult for Utto to fall into bad habits, introducing friction into things like frivolous online spending. This could be achieved in a number of ways, such as ‘locking’ certain apps, slowing scroll speed, burying them far from the ‘home screen’, or even introducing strong haptic feedback to illicit meta-cogntive monitoring (Manshad & Brannon, 2021).

When the stress is really on, the system gets creative, introducing rhythm into the environment through lighting in ways that have been shown to entrain neural oscillations (Charalambous & Djebbara, 2023). Such entrainment results in a resonance between brain, physiology, and environment, and is effective in influencing behaviour. The deliberate modulation of pattern frequency in environments, for example, can modulate the speed of people walking through a space (Djebbara et al., 2022). The system can also shift the intensity of light and colour to create atmospheric priming states, shifting an agent’s emotional experience (Canepa et al., 2023).^3^ Haptics can be deployed in similar ways, using soft textures, temperature, and rhythms to entrain breathing patterns effective for anxiety reduction (Shor et al., 2021), or modulating the frequency of vibration in haptic feedback systems to elicit different emotion responses. More speculatively, the system could use lighting, sound, and haptics to reflect Utto’s internal states (like heart rhythm), thereby putting him *within* his own body and training interoceptive accuracy, which can potentially ease symptoms of multiple psychological health concerns (Garfinkel et al., 2015; Quadt et al., 2021; Nord, 2022). Utto may even decide to wear a fairly unobtrusive device capable of inducing minor physiological changes, such as frisson, deployed to provoke specific interoceptive inferences, thereby inducing positive emotional experiences (Schoellar et al., 2019).

Importantly, much of this will be achieved through ASEs’ ability to simplify a problem space. However, one less obvious function of an ASE will be to temporarily and tactically *increase* the complexity of a problem space. Recent work has argued that the ability to increase complexity could have therapeutic potential, supporting well-being by disrupting overly powerful attractor states (which are hypothesised to underpin various forms of psychopathology (e.g., White & Hipólito, 2024). To take a simple example, consider how an ASE could subtly destabilise the kinds of habitual avoidance behaviours that can entrench episodes of depression, such as becoming socially withdrawn, and relying on the same repetitive routines of physical and mental inactivity. Interestingly, this places ASEs in line with accounts of the therapeutic potential of psychedelic therapies, which have been hypothesised to disrupt rigidly entrenched patterns of neural activity (Hipólito et al., 2023).

## Ambient smart environments and “classical” extended mind

In this section, we explore how ASEs fare in regard to classical TXM and its conditions. We see ASEs as one of the “exotic puzzle cases” anticipated in the original paper, and think that the exploration of ASEs in relation to classical TXM will tell us something interesting not just about ASEs but also about TXM itself.

### Sensorimotor interaction

A good place to start is with a recent, clear formulation of TXM, endorsed (at the time) by both Clark (2019), and Chalmers (2019):

A subject’s cognitive processes and mental states can be partly constituted.

By entities that are external to the subject, in virtue of the subject’s sensorimotor.

Interaction with these entities (Chalmers, 2019. Pp. 15).

The whole point of an ASE, however, is that the inhabitant doesn’t have to engage in sensorimotor interaction.^4^ As the above section described, the real functionality of the ASEs lies upstream of sensorimotor interaction. Why should we think that sensorimotor engagement is so crucial though? Chalmers (2019) introduces the stipulation to capture what is “strong and interesting” about TXM (pp. 15). The thought is this: some proposed (more science fiction) cases of cognitive extension involve parts of a person’s brain being replaced with an external silicon circuit, such as in Clark (2008). If we were to lose the ability to do mental arithmetic, and received a neural implant that restored that ability, it’s argued that accepting that silicon circuit as a proper part of our mind means accepting that minds can be extended. But Chalmers (2019), along with Farkas (2012), seems to think that these kinds of cases fail to capture what’s controversial about TXM. Chalmers points out that critics of TXM happily endorse such cases but want to reject a class of other cases. That class of other (rejected) cases are, Chalmers thinks, captured by them involving sensorimotor engagement, and so the stipulation is added not because cases that don’t involve sensorimotor engagement shouldn't count as extended, but because they count too easily.

Clark and Chalmers’ current view is that this explicit sensorimotor stipulation is too strong.^5^ We believe that the spirit of the sensorimotor stipulation can be preserved whilst also making room for interesting cases of extension in which there is no sensorimotor interaction. We believe that the core claim of TXM regarding sensorimotor interaction—the way that stipulation *should* be interpreted—is that the presence of a sensorimotor interaction does not create a barrier such that a cognitive process must always stop when a sensorimotor interface is crossed. As Chalmer’s points out, many cases that do not cross that line may be uninteresting or uncontroversial, and so not typically the subject of arguments favouring TXM. But we think that ASEs are a prime class of cases where the extension claim is both very interesting and does not involve sensorimotor interaction.

### Trust and glue

“Trust” refers to the automatic endorsing of, in Otto’s case, the information in the notebook. It also, arguably, refers to the fact that Otto, at some point in the past, consciously endorsed the entering of the information into the notebook (Clark & Chalmers, 1998. Pp. 17). Trust is taken to be a crucial condition of extension.^6^ Firstly, it seems wrong to count something as a part of *my* mind if I don’t trust it. But secondly because it’s taken to be a possible point of functional non-parity between internal and external resources. For example, Sterelny (2004) made the case that Otto’s notebook should not be counted as a part of his mind due to its comparable vulnerability, both to accidents (Otto dropping it into a puddle), or by deliberate sabotage (one of Otto’s many enemies inserts some false beliefs). The importance of such consideration is contested though, with Sterelny himself softening some of these worries about the notebook in later work (Sterelny, 2010).

In the case of ASEs, we can ask if the flow of information and affordances sculpted by the ASE feel like something that can be automatically endorsed. A wide body of work in human–machine interaction has cast trust as a “mutual predictability” between a human agent and a machine (Mayer et al., 1995; Lee & See, 2004; Montague, 2010; Hoff & Bashir, 2015; Schoeller et al., 2021). According to these accounts, although they vary in some details, fundamentally an agent trusts a system when that agent can model the consequences of actions involving that system with a high degree of accuracy, resulting in interactions with low degrees of operational uncertainty. As far as ASEs are concerned, their role in shaping our attention in ways that reduce the complexity of a task seems like prime candidates for functions automatically endorsed by the user. For example, having the ASE draft non-critical emails could become something I predict the system will do well.

But what about that other form of functionality, whereby the ASE increases complexity to destabilise rigid patterns. In these cases, the imperative of the system is to be, on some level, unpredictable. Imagine if Otto licensed an exciting but well-meaning friend to occasionally throw some curveballs his way via interventions in the notebook. In regard to ASEs, there is a degree to which this worry is only surface level referring simply to the degrees of freedom we perceive in the system; while we know that our smart kitchen might surprise us with new recipe suggestions, we trust that it won't surprise us with an arsenic-laced quiche. However, there is a sense in which ASEs could be a more serious threat to trust. While this isn’t quite the same notion of trust deployed in Clark and Chalmers (1998), we should consider the capacity of the system to operate in support of fulfilment of genuine preferences, which can often be nested, conflicting, or otherwise complex. Systems like ASEs could support loftier goals like personal growth and healing, but they could also pander to our lower-order preferences, thereby undermining a robust sense of personal agency (White, 2024). The relationship between highly adaptive personal technology and agency is a topic worthy of further work, but for now it’s simply worth recognising that even systems that are entirely predictable in their fulfilment of genuine preferences, could still pose a risk of harm.

One option to address this concern is by recognising that the dominant account of trust, based on ‘mutual predictability’ or ‘reliability’ might not be wholly suitable. Many philosophers have criticised this conception of trust as anaemic and offered more normatively thick conceptions of trust (Baier, 1986; Faulkner, 2017; Hollis, 1998; Stern, 2017). Adopting a less mechanistic account of trust will help us understand how an agent might trust a system like an ASE, even when it operates unpredictably and in ways that, temporarily at least, might make life more uncertain. Briefly, Kiran and Verbeek (2010) provide an account of trust in human-technology relations based on the confidence to “trust oneself to technology”(Kiran & Verbeek, 2010. Pp. 411). This account is premised on the idea that technology isn’t external to human experience, but rather that technologies constitute human subjectivity, in effect changing what it means to experience the world as human. This might mean more or less predictability or automatic endorsement, but is less about the performance of the technology in relation to a defined task, and more about how accepting we are of the shifts in phenomenology and identity. On this account, the notion of trust takes a meaning closer to what we understand the original meaning of “automatic endorsement”. One trusts an extended mind if one has no other choices than doing so. I trust my arm, not because it doesn't fail me, but rather because it is anchored to me and because I cannot conceive how it would be otherwise. It is in that sense that one “automatically endorses” an extended mind. The hard question is how can one endow an extended mind with such deep trust?

This is where the “glue” aspect becomes important. “Glue” refers to the way in which an extended mind such as Otto’s notebook becomes “a constant in Otto’s life”, with it being consistently consulted and the information being “directly available without difficulty” (Clark & Chalmers, 1998. Pp. 17). There are, arguably, two ways in which an extended mind comes to be. There are “palliative” cases where the extended mind fills a gap in the cognitive abilities of an agent (e.g., Otto’s notebook). In palliative cases the extended mind is glued through filling the gap left by the lack of a cognitive ability (e.g., Otto’s memory dysfunctions). There are also “prosthetic” cases where the extended mind replaces an existing cognitive ability. Prosthetic cases, arguably, lead to a higher level of trust than the palliative cases, as their adoption and “glueing”– the replacement of the existing ability– is driven by their recognised superiority and benefit. In prosthetic cases, the extended mind is glued through (i) making obsolete the use of onboard cognitive functions and (ii) making habitual/cultural/biological the use of the material, technique or technology that will constitute the extended mind. On the developmental time scale, this may look like losing the ability to navigate a city after starting to use a GPS. On the evolutionary time scale, this could be a niche construction process that makes a trait obsolete and replaces it (e.g., the lost ability to digest certain food through the acquisition of food processing techniques) (see Constant et al., 2022 for a discussion). ASE, if they are to be considered as extended minds, could become glued either as palliative extended minds or prosthetic extended minds. As palliatives, they can become glued through handling tasks that may otherwise be too stressful for users suffering from, say, anxiety issues. As prosthetics, they may become glued to through losing, say, the ability to write proper emails that would be replaced by the customary use of an AI email assistant. As a user would be living inside the ASE, it would likely exhibit very high degrees of accessibility and reliance. ASEs can’t be left behind somewhere or dropped, and when operational they do not need to be accessed in any intentional way. But Otto’s notebook, as well as the ubiquitous smartphone, can now be taken anywhere, and accessed on demand. ASEs are not like this, in the sense that the whole home can't be taken with us to the office or on holiday. This isn’t to say that the ASE can’t function while we’re away. For example, the system might monitor and adjust humidity levels in the greenhouse while we relax on the beach somewhere. Moreover, the functionality of the ASE could be extended in an impoverished fashion by downloading information to a set of smart glasses that can then overlay the information in augmented reality. What is clear is that in the case of ASEs, levels of “trust and glue” as outlined above, will be different between systems and users, and will likely fluctuate over short and long timescales.

### Transparency, bidirectionality, and speed

There are also constraints referring to the coupling between device and user. These are articulated in Kirsh (2019), as: phenomenological transparency, speed, and synchronous bidirectionality. Bidirectionality must, according to Kirsh, be fast and fluid, such that the changes are “synchronous.” For example, as one uses a pen and paper to jot down notes to form an idea, they form a “tight interactive loop” with the page where thought shapes writing and writing shapes the emergence of thought. Kirsh argues that adopting the synchronicity requirement for bidirectionality is an effective guard against the feared cognitive bloat (see Sec. 1.2). There’s no reason to think that an ASE could not operate in a synchronous, bidirectional manner, especially using the biofeedback features outlined earlier. And surely, the learning that ASEs undertake will help underpin this fluid bidirectionality; the more the system models a user’s preferences and intentions, the faster it will be able to shape affordances, making the case for extension (by these lights) increasingly plausible. As Clark (2003) notes: “The more closely the smart world becomes tailored to an individual’s specific needs, habits, and preferences, the harder it will become to tell where the person stops and this tailor-made, co-evolving smart world begins” (pp. 30). However, although ASEs will be capable of synchronous bidirectional coupling, much of their functionality will rely on processes and interactions unfolding diachronically. This potential for diachronic coupling will be addressed in later sections.

There is agreement among numerous theorists that cognitive extensions must be phenomenologically transparent (see Thompson & Stapleton, 2009; Kirsh, 2019; Wheeler, 2019).^7^ Kirsh (2019) argues that prosthetic limbs are “the least problematic of human extenders and therefore offer hints about what to expect of cognitive extenders” (pp. 131). This notion of transparency draws on work by Merleau-Ponty describing how blind people apprehend the world *through* the cane, never experiencing the cane as separate from themselves. In the case of cognitive extensions, most of us will be familiar with software or operating systems that we hate using, because they feel clunky. Here it feels like our cognitive efforts are directed as much at the software as the task at hand. Alternatively, when software is well designed, it can feel like a clear window we act through. ASEs are, potentially, exemplary transparent technology. But this depends on how we interpret ‘transparency’, as in most cases, it seems to be taken to apply to tasks involving intentional motor action (i.e., a blind person using a cane, or a skilled artist using a sketch pad). ASEs don’t involve these kinds of intentional motor interactions, but their functionality is transparent in the sense that we don’t act on it, but, in a sense, we do act *through* that functionality. This kind of transparency, coupled with their adaptivity, likens them to systems Clark (2003) describes as “pseudo-neural”, in the sense that many of the operations of neural circuits are transparent and require no conscious deliberate effort (think about the fact that I hardly deliberate over every movement of my fingers in writing this sentence) (Clark, 2003. Pp. 45).

On the other hand we might think that this kind of transparency should make us suspicious, as it disrupts our ability to individuate cognitive functions that are putatively extended. As mentioned above, Kirsh (2019) suggests that cognitive extensions must have a “bodily component” (pp.131). In the case of Otto’s notebook, a clearly delineated system manifests through sensorimotor interaction, extending a definable function. The ubiquity of the ASE and the range of different applications described seem more like a general and constant mediation between user and world in a way that makes it difficult to pin down specific functions (See, e.g., Kiran and Verbeek, 2010a; b; Aydin et al., 2019).

We think that the picture that emerges when ASEs are examined within the context of the constraints of classical TXM is somewhat unclear. Although we don’t view the lack of sensorimotor engagement in the case of ASEs as precluding them as cases of extension, it’s plausible that the ubiquity and automaticity make it difficult to pin down the precise functions being extended. As many authors note, literature on TXM has almost always focused on the completion of a specific task, thereby highlighting a specific and well-defined cognitive function (Aagaard, 2021; Bruineberg & Fabry, 2022; Slaby, 2016). Although they are critical of this bias, highlighting and addressing the absence of work on non-task related cognition, the role of ASEs as supporting such a broad array of functions makes it difficult to apply the above conditions clearly.

## Active inference and the extended mind

### Active inference

Frameworks in theoretical neuroscience have reinvigorated discussions around TXM. The active inference framework (AIF) sees agents as embodied uncertainty minimisers, instantiating a generative model of self and environment (Friston, 2010; Hohwy, 2013; Clark, 2015; Parr & Friston, 2017; Parr, Pezzulo & Friston, 2022). A recognition density encodes sets of expectations (priors), generating predictions about the causes of sensory states (e.g., that crash downstairs is just the wind and not a burglar), and about the most effective action strategies (e.g., I should probably go and check anyway). Under the AIF, an agent’s imperative is to minimise the long-term uncertainty associated with its own sensory observations (prediction errors), which can be done in three ways: first by updating predictions to better match the data, second by changing the data that is being perceived through acting on the sensorium (e.g., moving your eyes), and third, indirectly, by acting to bring the world in line with expectation– also referred to as cognitive niche construction (Constant et al., 2018; Constant et al., 2022; Bruineneber et al., 2018). By occupying the states that are ‘expected’ (given the agent’s specific phenotype and history), the agent keeps itself healthy and alive.

Prediction errors, however, are not all equal. In a world as noisy, some sets of errors are more salient and reliable than others, and agents must be able to separate signal from noise. The modulation of salience and reliability is achieved directly, downstream using a mechanism of second-order prediction known as ‘precision weighting’ (Friston, 2010; Hohwy, 2013; Clark, 2013a, 2013b, 2015), and indirectly, upstream through the mechanism of cognitive niche construction whereby irrelevant sensory inputs are made unavailable, through environmental modification. Consider crossing a busy road in a major city. Our experience is a cacophony: tourists chattering, billboards advertising multi-vitamin pills, flashing lights, and road signs. Given our strong expectation of crossing the road safely and our model of the regular causal dynamics of busy intersections, we can expect higher precision on certain input streams. The system effectively turns down the ‘volume’ on the billboard and the tourists, and ups the ‘volume’ on the sound of approaching buses. On a foggy day, precision on all visual information can be reduced, entraining exploratory action like finding a safer crossing. We could, collectively, also implement upstream niche construction strategies, such as deciding to adopt urban planning rules that prohibit invasive forms of marketing in certain areas requiring focus. Thus, precision weighting and niche construction instil predictive systems with a high degree of crucial contextual flexibility at multiple temporal scales, from low-level neurocognitive to high-level social organisation.

### Allostasis

A crucial element here is that agents don’t solely aim toward minimising present prediction error; we plan and act to minimise *expected* uncertainty over the long-term (Parr and Friston, 2017; Kiverstein et al., 2020). This focus on expected uncertainty speaks to the theoretical unification of actions both pragmatic and epistemic under uncertainty minimisation: both pulling out an umbrella and watching the week’s weather forecast are folded under actively inferring that we remain dry. In order to undertake this complex concert of temporally interlocking action policies, agents are hypothesised to be sensitive to changes in the rate of error reduction over time relative to expectation, known as ‘error dynamics’ (Joffily & Coricelli, 2013; Van de Cruys, 2017; Kiverstein et al., 2020). Error dynamics holds that changes in the rate of reduction are fed back on the subjective level as bodily affect, and this bodily affect influences and reflects changes in precision weighting, thereby updating expectations in an ongoing way sensitive to how the agent is doing. The totality of this picture is of an agent attuned to a range of both epistemic and pragmatic affordances, constantly tuning the precision of policies that reflect the best predicted trajectories of expected uncertainty unfolding over multiple overlapping timescales, the success and failure of which is constantly fed back through affectivity.

An inability to manage uncertainty is linked to stress and illness. The anticipatory actions that underpin our ability to manage expected error reflect *allostasis*, which describes the adaptive changes that respond to external perturbation, maintaining homeostasis (Sterling & Eyer, 1988; McEwen, 1998; McEwen & Wingfield, 2003; Barrett, et al. 2016). Being in an unresolved allostatic state demands that the body operate beyond its typical physiological setpoints: for example, the changes in physiology (like rapid breathing) accompanying a perceived threat. Typically, these changes are short-lived responses to external stressors, but in cases where there's a breakdown in optimal agent-environment dynamics, these periods may be prolonged, resulting in damage to the body's vital underlying processes and systems (Koob & Le Moal, 2001). The effects of staying within an unresolved allostatic state have been termed *allostatic load*, and negatively impact many vital systems including the immune system, endocrine system, and cardiovascular system (Barrett et al., 2016; Guidi et al., 2021; Koob & Le Moal, 2001), and allostatic load is associated with severe negative health outcomes (Arnaldo et al., 2022; Rabey & Maloney, 2022).

### Active inference and TXM

Our goal in the remainder of this section is to highlight the contributions AIF researchers have made to TXM, and in the next section we then apply this work to ASEs. The potential of the AIF to expand classical TXM is articulated in Constant et al. (2022), who argue that the AIF provides a “formal apparatus to make progress” in thinking about different cases of cognitive extension. The claim is that the formalisms of the AIF vindicate the parity principle by describing both the process of surprise minimisation for brain-bound processes *and* processes involving extra-bodily resources. In other words, prediction error minimisation describes equally body-bound dynamics *and* actions involving material environmental features. Constant and colleagues (2022) say nothing about the conditions of TXM discussed earlier, but they are explicit in their objective to “provide future researchers with a formal apparatus to make progress in these debates by showing how the varieties of claims on extended cognition may be formally expressed” (pp. 388).

Constant and colleagues’ aim is to show how an AIF formulation of niche construction, one that the authors term ‘extended active inference’ (EAI), casts cognitive niche construction as a form of cognitive “uploading” or “offloading”, of the kinds described by the canonical cases of TXM (i.e., the use of artefacts like notebooks and smartphones).^8^ Although in their view cognitive niche construction goes beyond strict cases of TXM, they write that “over developmental time, smartening the world through cognitive niche construction operates through processes akin to…the extended approach to cognition” (pp. 387). Adopting the AIF as a lens through which to view these activities (leveraging the niche as an externalised, shared generative model) allows us to qualify cases typical of TXM as genuine cognition. Under EAI, *all* cases of cognitive offloading or uploading, whether it's treading a desire path used by subsequent generations, or the short-term use of a computer, can be formalised as prediction error minimisation driven through cognitive niche construction (Constant et al., 2022. Pp. 377). Here, uncertainty minimisation, acting as a shared computational currency, unites cognitive processes underlying perception and action with material-involving practices (Constant et al., pp. 377).

One way of thinking about this EAI picture, and how it shifts our perspective on TXM, is to see how *expected* changes to the rate prediction error reduction can help select cognition-action cycles that include external structures and resources. According to Constant and colleagues, internal salience mappings can dictate the use of external resources, such as when I check my phone as the quickest way to know the time of a flight departure, and cases where those salience mappings themselves can be externally cued, such as setting an alarm to remind myself to check the departure time (Constant et al., 2022. Pp. 389). In other words, given a certain task, the brain has no prejudice in estimating that the best rates of error reduction will rely on external tools. This picture is developed to a great extent by Clark (2015, 2016, 2017, 2022). Clark argues that understanding cognition through the AIF casts agents as constantly “knowledge budgeting”, wherein the minimising of relevant prediction error includes marshalling whatever material resources happen to be readily available for efficient use (Clark, 2022. Pp. 4). The recruitment of external tools is a natural part of the brain’s core strategy for minimising prediction error.^9^

### Recruitment puzzles

One way to appreciate the impact of the AIF, is to see that classical TXM lacked any specific notion of brain function and relied on coarse-grained functionalism. In short, the parity principle entailed that questions around extension be predicated upon the potential for a functional isomorphism between biological and non-biological vehicles of cognition (Constant et al., 2022). This had the benefit of helping us overcome our chauvinism toward bio-internal structures, thus broadening the mind both literally and figuratively. But it also left open the question of how the brain-body system ‘decided’ when, where, and how to recruit bio-external hardware. This was known as the “recruitment puzzle” (Clark, 2008). Consider a student attempting to solve a difficult maths problem, but having access to multiple tools. Classical TXM with its coarse-grained functional approach can describe the way that picking up, say, a calculator, forms an extended cognitive system based on the parity principle, but the recruitment puzzle remains: how can this coarse-grained functionality describe *how* one resource is recruited and not any other, and what cognitive process drives the recruitment? In light of active inference, however:

The answer to the recruitment puzzle is now clear. Predictive brains constantly estimate.

The extent to which taking an action…will reliably reduce uncertainty in ways that help us approach our goals (Clark, 2022. Pp. 6)

A critical part of this answer is that predictive systems improve their models of the world not just by updating expectations, but also by reducing the complexity of the model, the world, or uncertain generative processes (Hohwy, 2013; FitzGeralds, Dolan & Friston, 2014; Clark, 2015; Constant et al., 2018; Constant et al., 2022). In the context of recruiting non-bodily cognitive resources, precision weighing allows the system to flexibly select transient assemblages that represent the strategies the system is most confident deliver the best balance between accuracy and complexity. As Clark (2015) argues, cognitively complex agents with temporally thick generative models can learn over time strategies that utilise known environmental features that are just as apt for precision-based selection as any internal routine (Clark, 2015). By understanding these assemblies in this way they increasingly appear less amenable to the strict conditions explored above (Sec. 1.2): the best combination of accuracy and complexity right now might be ill-suited for a task five minutes from now, and levels of trust and transparency can, therefore, shift moment-to-moment.

### Shifting boundaries and process ontology

Regarding how this picture fits with classical TXM, Clark (2017) suggests that the boundaries of self-evidencing systems like us are constantly shifting to accommodate different assemblies of internal and external components that form part of our prediction error-minimising machinery.^10^ As such, he calls for us to adopt a “process ontology” (Dupré, 2014) approach to identifying what counts, at any given time, as part of a cognitive system (Clark, 2017. Pp. 13). This is opposed to the kind of fixed state ontology that characterises the metaphysical conception of material objects, and seems implicit in much classical TXM literature. A process ontology better captures the nature of a system that “builds and rebuilds” its own boundaries in real-time through temporary precision mappings, characterised by “mutability and multiplicity” (Clark, 2017. Pp. 7). An implicitly assumed state ontology is, arguably, accountable for some of the worries that dogged the classical version. For example, arguments about whether external resources were to be understood as constituting part of a cognitive system, or ‘merely’ acting causally on the internal (“truly cognitive”) machinery, led to accusations against TXM theorists of committing the ‘causal-constitutive (C–C)’ fallacy (Adams & Aizawa, 2001, 2009, 2010). The argument is that proponents of TXM unjustifiably mistake elements which are causally relevant to a system to be constitutive of that system. For example, while a piece of marble materially constitutes the statue *David*, it does not cause *David.* Conversely, while the fire alarm causes me to evacuate, the fire alarm does not constitute my evacuation (see Kirchoff, 2015).

In response, an alternative account of constitution—diachronic constitution—has been proposed (Kirchoff, 2015; Kirchoff & Kiverstein, 2019). Proponents of the diachronic account of constitution argue that the C–C fallacy is based on a mistaken assumption that cognitive processes are amenable to the same conception of constitution as material objects. Instead, it is argued that cognitive processes are different because they are, first, made up of interlevel dependence relations, and second, because they are temporally multifaceted, with constitutive subprocesses (synaptic firing) unfolding over different timescales to the higher-level process (recalling the location of a museum) (Kirchoff, 2015). These differences are at the core of the distinction between process and state ontology. Explaining how what we experience as water is diachronically constituted by a temporal flow of ongoing multi-level processes, Ross and Ladyman (2010), describe how:

It makes no sense to imagine it having its familiar properties synchronically. Rather, the water’s wetness, conductivity, and so on all arise because of equilibria in the dynamics of processes happening over short but non-negligible time scales at the atomic scale. From the point of view of any attempted reductive explanation, the kind water is not held by physicists to be ‘constituted’ as opposed to ‘caused’ because it is not a substance in the classical metaphysical sense (Ross and Ladyman 2010, 160)

Thus, when Clark argues for a process ontology of extended cognitive systems, it implies a notion of diachronic constitution. In a nutshell, cognition is more like a river than it is like a chunk of marble—the interesting characteristics are underpinned by ongoing multi-level, temporally extended processes. This shift toward a process ontology and diachronic constitution is supported by the AIF, which emphasises the multi-layered temporality of our actions and strategies (Kirchhoff & Kiverstein, 2019). The generative models of active inference are temporally deep and hierarchical, with upper levels tracking slower timescales and lower levels tracking faster causal regularities. The rapidly unfolding predictions at lower levels, pertaining to fast perception–action loops, ground and update the more slowly unfolding expectations at higher levels. The temporal thickness of models is what allows human agents to engage in the allostatic control outlined earlier. When viewed through the AIF, we see more clearly how cognitive processes necessarily occur across timescales, where fast processing at one level partly constitutes processes unfolding more slowly at other levels (Kirchhoff & Kiverstein, 2019).

The goal of the foregoing paragraphs has been to show how the AIF has provided motivation and means for reimagining TXM in a different way: first, the formalisms of the AIF vindicate the parity principle, moving it from a more coarse-grained functionalism, to a finer-grained computationally unified perspective. This provides a surer footing for TXM theorists, and removes the “cost of going coarse grained” as Sterelny (2010) puts it (pp. 471), which is a reduction in richness of the overall theory. This TXM is one that is more fluid, characterised by a process ontology of rolling assemblies (of biological and material vehicles). This should soften the instinct to apply fixed boundaries for the definition of extended cognitive systems. This isn’t because boundaries become unimportant in specific circumstances or in regard to important questions, but because the application of strict boundaries becomes more difficult as these processes unfold over time and across different scales. The temporal dynamics essential to understanding the AIF describe a different kind of constitution for cognitive processes that lends itself to diachronic couplings that assemble and dissipate over time. Thus, the AIF provides the theoretical tools for cashing out these conceptual shifts, providing a view of brain function that is hierarchical and transient, where recognition of salience is achieved through temporary webs of precision weighting estimates. The view of the cognitive agent that emerges from the AIF, then, is one in which boundaries are characterised by a healthy degree of ambiguity and inconstancy.

## Active inference extended minds, and ASEs

### ASEs: extended allostatic control systems

This emerging vision of TXM seems to be more in the spirit of Clark (2017), which casts human agents *qua* cognitive systems as fundamentally “meta-morphic.” It also seems better poised to capture that diverse, fast functionality of ASEs. While Otto’s notebook serves a relatively fixed functionality and requires an intentional engagement, new technologies—if we are inclined to think of them as extensions—will necessitate a faster and more fluid picture of cognitive extension. The ubiquity of the ASEs functionality and the range of different applications described seem more like a general and constant mediation between user and world in a way that makes it difficult to pin down specific functions (See, e.g., Kiran and Verbeek, 2010; Aydin et al., 2019).

The view of ASEs we present here is one of a general allostatic and epistemic support system, the multifacetedness and fluidity of which means that its fulfilment of certain conditions and constraints will never be stable. The literature on TXM is testament to our proclivity to pick out specific cognitive functions. However, we should note that the original extended mind paper was a call to accept a broader material base for our mental lives generally, and wasn’t meant to restrict our application to well-defined functions (Clark & Chalmers, 1998). Keeping this in mind should dampen our instinct to decompose the role of ASEs; we want to capture that combined functionality rather than any one strand. Moreover, the AIF casts all cognitive behaviours, whether that's remembering the location of a museum or writing a novel, as a process of prediction error minimisation.

The second difficult aspect of ASEs is their ambience. We might think, for example, about the way that many background features of our environments passively support cognition all the time. Clark (2008), for example, wonders whether the rhythmic lashing of rain on an office window, conducive to concentration, should be counted as part of my assembled cognitive substrate when writing (pp. 130). Clark concludes that no, the rain should not be counted as it isn’t “selected and maintained” as part of my system. But the ambience of an ASE is different. Consider again the example of Utto, and the ways that ambient lighting in the walls and haptics in the work chair use rhythms to entrain responses directly relevant to Utto’s needs in real time, based on up to date information about his internal states. Thus, we can distinguish between the passivity of general environmental features which, under certain circumstances, might support us in whatever task we’re doing, and the kind of adaptive functionality of a system like an ASE. The “passive” features of ASEs are stronger candidates for extension because they are *maintained and selected*, and in those moments trusted and closely coupled to Utto. But exactly which functions should count at any given time as part of an extended system could fluctuate as the user switches between tasks.

An interesting path forward is to think of ASEs as extended allostatic systems. The function of the ASE, on this view, is to extend an agent's biological capacity for avoiding prolonged allostatic states (i.e., states that demand operation beyond the body's physiological homeostatic setpoints), which, under the AIF, includes both pragmatic policies and epistemic policies. The ASE serves as an extension to these pragmatic and epistemic capacities for anticipation and adaptation. The system’s immense capacity for learning extends the epistemic demands of allostasis, while its pragmatic functions reduce the energy demands of constant adaptation during times of high stress. We chose to conceptualise these systems as extended allostatic control for several reasons. Firstly, to be clear, we take allostatic control to be a truly *cognitive* process in that it is maintained through adaptive planning and action and the role that these operations play in the ongoing resolution of uncertainty.^11^ Moreover, extended allostatic control captures the task diversity of ASEs; from supporting emotion regulation to maintaining a household budget, the diverse functionality of the ASE is, ultimately, working in the service of avoiding prolonged states of stress. In the philosophical literature, we might strongly associate this with the maintenance of an “optimal grip” (Merleau-ponty, 1962/2012; Bruineberg & Rietveld, 2014; Kiverstein et al., 2020), but the notion of “grip” is “primarily phenomenological”, describing a component of subjective experience rather than an underlying process (Bruineberg & Rietveld, 2014). Extended allostatic control also speaks to the anticipatory function of ASEs, manifest across multiple interlocking timescales; the upstream functionality of an ASE may unfold over more than just the present moment, learning to predict, anticipate, and mitigate periods of stress, and sculpt the ongoing affordance field accordingly. This functionality overlaps neatly with biological allostatic control.

The role of attention in allostatic control is crucial, and can be brought into sharp relief with recent insights from the AIF. Deane et al. (2024) describe a hypothesised system of ‘adaptive narrative control’ based on the flexible and subpersonal deployment of precision weighting to avoid negatively valenced affective states. The key insight here is that in order to maintain good allostatic control, the generative model need not deploy precision in a strictly truth-tracking way, but that the generative model includes the “pragmatic implications” of specific attentional states (Deane et al., 2024. Pp. 8). Such a system is described as a consequence of intelligent agents gaining the requisite sophistication to anticipate and model future contingent affective states (e.g., if I pay attention to *x* I will feel bad). Think, for example, about a person experiencing financial difficulty that, for the time being, they have little or no means of remedying. The person may know that it’s a good idea to check their bank balance and credit card statements, and yet continue to spend money and fail to track the reality of their situation. These kinds of avoidant behaviours and forms of denial are accounted for on this view by the adaptive narrative control system allocating precision in a way that avoids the allostatic stress response of facing financial reality (Deane et al., 2024). Of course, we don’t want ASEs that disconnect us from reality in problematic ways, but it’s easy to imagine systems that extend this kind of adaptive narrative control, carefully patterning our attention, shaping the field of affordances, such that prolonged periods of stress are avoided. We think, therefore, that conceptualising the functionality of ASEs as a form of highly adaptive extended allostatic control system combining learning, passive adaptation and active intervention, does justice both to the potential power of ASEs, and to the flexibility of a process-based TXM.

### Concerns and future questions

In this final subsection we address what we think could be a natural worry about the account we’ve provided. In advocating for this more fluid approach to TXM, pushing for a TXM that can accommodate the great swirl of shifting temporary couplings characterised by varying degrees of trust and transparency, might threaten to leave us unable to articulate a bounded agent in contexts that are important. In a nutshell, the worry here might be that in adopting a process-based TXM with its multiple shifting boundaries, we essentially do away with the notion of a bounded agent altogether.

It’s crucial to make clear that this is not the case, and does not follow from the account provided above. We aren’t at all advocating for an abandonment of any meaningful notion of a self as separate from the environment. Rather than obliterating boundaries, we take the version of TXM outlined here and applied to ASEs, to powerfully demonstrate the view articulated in Clark (2017), namely that the boundary between agent and environment really is constantly in flux; there is “neither a unique nor a stationary boundary” (Clark, 2017. Pp 6), but there will always, within a specific context, be a boundary relevant to our concerns. Emerging technologies will, however, increasingly bring to our attention the way that the persisting agent cannot be captured through a single *stationary* boundary. When the need does arise for asking where, at a specific moment in time, the boundary of the agent was (in the case of an assault, for example), we can fall back on the relevant sensible conditions. The specific legal or moral considerations in play should guide our intuitions about the most relevant considerations. As Kirsh (2019) puts it: “As cyborg couplings proliferate, the voice of justice will have to clarify things…Law is the arbiter, but scientists will be the expert witness” (pp. 141). The adoption of multiple shifting boundaries also need not re-raise concerns about “cognitive bloat”, as a process-based TXM facilitates the drawing and redrawing of boundaries in an ongoing way as specific task-relevant resources change. Considering a person entering a library, we aren’t forced to think in static terms about where in the library the boundary of the agent’s mind really sits. Instead we can consider which resources are being recruited for prediction error minimisation which may change over time as the agent consults a computer station, pores over one book, and then two together, uses a pencil to make notes, and so on. Finally, another consideration for thinking about shifting selfhood and TXM would be to consider the broader political and economic contexts in which material and technological culture arises. While agents will (hopefully) freely choose how their engagements with ASEs happen, this isn’t true for so much of our embedded and scaffolded endeavours, and considering the wider influences that shape our engagements, and the interests in play, could help us think about relevant boundaries and the forces that shape them (see, e.g. Slaby, 2016).

## Conclusion

We began in Section One by reviewing the classical version of TXM and exploring in detail some of the proposed conditions on extension. In Section Two we outlined the unique functionality of ASEs, drawing on work that casts them as a ‘meta-affordance’, acting upstream of our sensorimotor engagements to shape our affordances in ways that support general wellbeing. In Section Three we assessed the status of ASEs as extension by applying the classical conditions, and found that the picture that emerges is somewhat unclear; the peculiar functionality of ASEs seems to evade the stringent application of some conditions. In Section Four we introduced the AIF, and then explored how it should change the lens through which we think about TXM. We first saw how the formalisms of the framework vindicate the parity principle, shifting it from coarse-grained functionalism to a reflection of the shared computational imperative of prediction error minimisation. Next, we saw how the AIF necessitates a notion of cognitive processing as unfolding across multiple interlocking and nested timescales, and that this suggests a form of diachronic constitution and process ontology, as opposed to the state ontology and synchronous notion of constitution that have often been assumed to apply to TXM. In the final section, we applied this new fluid process ontology account of TXM to ASEs, arguing for an accommodation of ASE functionality as extended allostatic control.

Ned Block’s assertion that TXM was false in 1998 but true in 2019 reveals something fundamental about any theory that aims to capture the intimate relationship between mind and technology. Technological conception and design is endlessly changing, new developments can be surprising and even revolutionary, and the emergence of new technologies might always challenge the assumptions of our established frameworks. The running debates between classical TXM and its rivals, often with a strong emphasis on strict conditions and fixed boundaries, are ill-suited to account for a future in which people are increasingly enveloped by ubiquitous, adaptive, and highly individualised technological environments that support multi-timescale functions. Systems that will impinge on many aspects of a user’s cognitive worlds, from sending emails to their long-term mental health, are difficult to capture using the tools of classical theories. We’ve argued here that this new approach, made possible by the AIF, allows for a principled move away from static boundaries, strict conditions, and long-held dichotomies between these different approaches. While the scaffolded approach retains a historical emphasis on developmental and intergenerational timescales, learning and evolution, the AIF allows for a deep continuity with TXM. As emerging technology spectacularly amplifies human cognitive capacities, the approach afforded by the AIF will be increasingly appropriate.

## Footnotes

1 Throughout this paper we will typically refer to cognitive processing, rather than mental states. Although the original Clark and Chalmers paper (1998) distinguishes between cognitive processes and “truly mental states”, later work on the extended mind tends to treat “mind” and “cognition” as largely interchangeable (see, e.g., Wheeler (2019)).

2 We use smart here in its now largely colloquial usage, to refer to technology that exhibits anticipatory and adaptive capability through the use of algorithms and machine learning.

3 “Atmosphere” here refers to an intuitive notion used by architects to describe “the essence of affective qualities” present in the material space, that may be influenced by features such as lighting and colour (see Canepa et al., 2023).

4 Insofar as the ASE’s functionality doesn’t show as an affordance to be intentionally engaged with through motor action. There is, of course, a more minimal sense of sensory interaction in that the ASE is sensing and monitoring aspects of the agent, or in using sensory flows to entrain behaviour. Thanks to an anonymous reviewer for helping us to make this clear.

5 This current view, with its softening of the sensorimotor stipulation, was established during conversations at the 25th anniversary of the Extended Mind conference held in Boulder, Colorado, August 2023.

6 It is interesting that a failure of trust is widely agreed to preclude genuine cognitive extension. However, failure of trust is consistent with material scaffolding (see forthcoming special issue of Topoi titled “Scaffolding Bad: Varieties of Situated Cognitive Harm”, edited by Spurrett, Colombetti, & Sutton (2024).

7 Although this agreement is by no means universal, with some making persuasive arguments that phenomenal transparency is neither necessary nor sufficient (See Smart et al., 2022).

8 Contant et al. (2022) define cognitive offloading as the restructuring of material space to minimise the internal energy costs associated with a cognitive task, such as rearranging ingredients for a complicated dish on the kitchen top in the order they are to be used. This is to be contrasted with cognitive “uploading” wherein a material device like a smartphone or computer takes on the cognitive labour itself, such as in the case of a calculator doing long division.

9 Just how natural a process this is can be seen in studies that show how quickly the brain accommodates the uncertainty minimisation value of basic tools in Macaques, with neurons quickly re-representing objects as “near” or “far” depending on the reach capabilities of available tools (see: Berti & Frassinetti, 2000).

10 Although we want to avoid a lengthy discussion here, it is worth noting that the AIF has the mathematical and conceptual tools to account for systems with shifting boundaries. ‘Markov blankets’ serve to delineate and describe the boundaries between the internal states of the system and its external states. Markov blankets are not fixed or permanent, and a system can be comprised of multiple nested blankets (for discussion see: Friston, 2013; Clark, 2017; Hohwy, 2017; Kirchoff et al., 2018; Van Es and Kirchoff, 2021; Bruineberg et al., 2022).

11 Thanks to one anonymous reviewer for raising this point of clarification.
